# The impact of unexpected intensive care unit admission after cancer surgery on long-term symptom burden among older adults: a population-based longitudinal analysis

**DOI:** 10.1186/s13054-023-04415-8

**Published:** 2023-04-25

**Authors:** Bourke W. Tillmann, Julie Hallet, Rinku Sutradhar, Matthew P. Guttman, Natalie Coburn, Tyler R. Chesney, Jesse Zuckerman, Alyson Mahar, Wing C. Chan, Barbara Haas, members of the REcovery after Surgical Therapy for Older adults REsearch –Cancer (RESTORE-C) group, Amy Hsu, Douglas Manuel, Frances Wright, Dov Gandell, Ines Menjak, Lesley Gotlib-Conn, Grace Paladino, Pietro Galuzzo

**Affiliations:** 1 Interdepartmental Division of Critical Care, University of Toronto, Toronto, ON Canada; 2 Department of Critical Care Medicine, Sunnybrook Health Sciences Centre, 2075 Bayview Avenue – Room D108, Toronto, ON M4N 3M5 Canada; 3 Institute of Health Policy, Management, and Evaluation, University of Toronto, Toronto, ON Canada; 4 Department of Surgery, University of Toronto, Toronto, ON Canada; 5 Department of Surgery, Sunnybrook Health Sciences Centre, Toronto, ON Canada; 6 ICES, Toronto, ON Canada; 7 Clinical Evaluative Sciences, Sunnybrook Research Institute, Toronto, ON Canada; 8 Dalla Lana School of Public Health, University of Toronto, Toronto, ON Canada; 9 Department of Surgery, Unity Health, Toronto, ON Canada; 10 Department of Community Health Sciences, University of Manitoba, Winnipeg, MB Canada; 11 Ottawa Hospital Research Institute, Ottawa, ON Canada; 12 Sunnybrook Health Sciences Centre, Toronto, ON Canada; 13 Sunnybrook Research Institute, Toronto, ON Canada

**Keywords:** Neoplasm/surgery, Critical care, Older adults, Recovery of function, Quality of life

## Abstract

**Background:**

Older adults are at high-risk for a post-operative intensive care unit (ICU) admission, yet little is known about the impact of these admissions on quality of life. The objective of this study was to evaluate the impact of an unexpected post-operative ICU admission on the burden of cancer symptoms among older adults who underwent high-intensity cancer surgery and survived to hospital discharge.

**Methods:**

We performed a population-based cohort study of older adults (age ≥ 70) who underwent high-intensity cancer surgery and survived to hospital discharge in Ontario, Canada (2007–2017). Using the Edmonton Symptom Assessment System (ESAS), a standardized tool that quantifies patient-reported physical, mental, and emotional symptoms, we described the burden of cancer symptoms during the year after surgery. Total symptom scores ≥ 40 indicated a moderate-to-severe symptom burden. Modified log-Poisson analysis was used to estimate the impact of an unexpected post-operative ICU admission (admission not related to routine monitoring) on the likelihood of experiencing a moderate-to-severe symptom burden during the year after surgery, accounting for potential confounders. We then used multivariable generalized linear mixed models to model symptom trajectories among patients with two or more ESAS assessments. A 10-point difference in total symptom scores was considered clinically significant.

**Results:**

Among 16,560 patients (mean age 76.5 years; 43.4% female), 1,503 (9.1%) had an unexpected ICU admission. After accounting for baseline characteristics, patients with an unexcepted ICU admission were more likely to experience a moderate-to-severe symptom burden relative to those without an unexpected ICU admission (RR 1.64, 95% CI 1.31–2.05). Specifically, among patients with an unexcepted ICU admission the average probability of experiencing moderate-to-severe symptoms ranged from 6.9% (95 CI 5.8–8.3%) during the first month after surgery to 3.2% (95% CI 0.9–11.7%) at the end of the year. Among the 11,229 (67.8%) patients with multiple ESAS assessments, adjusted differences in total scores between patients with and without an unexpected ICU admission ranged from 2.0 to 5.7-points throughout the year (*p* < 0.001).

**Conclusion:**

While unexpected ICU admissions are associated with a small increase in the likelihood of experiencing a moderate-to-severe symptom burden, most patients do not experience a high overall symptom burden during the year after surgery. These findings support the role of aggressive therapy among older adults after major surgery.

**Supplementary Information:**

The online version contains supplementary material available at 10.1186/s13054-023-04415-8.

## Introduction

Older adults account for more than 50% of all new cancer diagnoses [1, 2]. With improvements in cancer therapies and advances in perioperative care, older adults are increasingly being offered aggressive surgical intervention as part of their cancer treatment [3–5]. Nonetheless, they remain at high-risk for post-operative complications, including admission to an intensive care unit (ICU) [6–8].

While ICU care may be lifesaving, older adults surviving critical illness are at risk for loss of independence, cognitive decline, and decreased quality of life, a sequala commonly referred to as the post-intensive care syndrome [8–18]. Many clinicians fear that post-operative ICU care may result in outcomes inconsistent with older adults’ wishes, including admission to a nursing home or ongoing support to perform activities of daily living [19–21]. However, key findings complicate decision-making following major cancer surgery. First, patients undergoing cancer surgery are highly selected and have better outcomes than would be expected from the general critical care literature [8]. Second, older adults who survive critical illness appear satisfied with their clinical outcomes, despite an increase in disability [22]. Third, among older adults there is limited information regarding the patient-reported experience after a post-operative ICU admission. This lack of understanding of the patient’s perception of their outcomes can result in communication pitfalls and lead to treatment plans inconsistent with their values [23–25].

To address this knowledge gap, we evaluated the patient-reported symptom burden among older adults who experienced an unexpected ICU admission after high-intensity cancer surgery, as compared to those who did not experience an unexpected admission.

## Methods

### Study design and setting

This was a population-based cohort study of older adults who underwent high-intensity cancer surgery in Ontario between 2007–2018 and survived to hospital discharge. Ontario is Canada’s most populous province (population 14.2 million), containing 217 acute care hospital sites of which 14 are regional cancer centers (RCCs) [26, 27]. All medically necessary services are funded by a public, single payer system with standardized reimbursement rates. This study was approved by the Sunnybrook Health Sciences Centre Research Ethics Board.

### Data sources

Data were derived from administrative datasets held at ICES [28–30]. These datasets were linked using unique encoded identifiers and analyzed at ICES. Details of the datasets are available in the Online Supplement (Additional file 1: eTable 1).

### Study population

We included patients ≥ 70 years of age with a newly diagnosed gastrointestinal, genitourinary, or bronchopulmonary cancer between January 1, 2007, to September 30, 2018, who underwent high-intensity cancer resection within 90 days before or 180 days after diagnosis, survived to hospital discharge, and were seen at a RCC at least once during the year after surgery. Malignancies were identified using International Classification of Diseases 10th Edition Oncology codes and procedures identified using Canadian Classification of Health Intervention codes (Additional file 1: eTable 2). High-intensity resections included lobectomy, pneumonectomy, esophagectomy, gastrectomy, enterectomy, colectomy, hepatectomy, pancreatectomy, adrenalectomy, nephroureterectomy, radical cystectomy, and prostatectomy [31].

Patients living in a publicly funded nursing home prior to surgery were excluded. Additionally, patients who had a cancer diagnosis within the previous 5 years or two or more cancer types diagnosed on the index date were excluded, as the presence of additional cancer diagnoses likely impacted the decision to proceed with surgery and/or post-operative outcomes. Patients with at least one patient-reported symptoms assessment recorded within 12 months of their surgery were retained for the primary analysis.

### Exposure

The exposure of interest was an unexpected post-operative ICU admission during the index hospitalization, defined as an ICU admission on any day other than the day of surgery, or any ICU admission associated with mechanical ventilation [8]. As it is common for patients undergoing high-intensity surgery to be admitted to an ICU post-operatively for routine monitoring, ICU admissions occurring on the day of surgery without mechanical ventilation were considered planned/expected admissions. ICU admissions were identified using previously validated algorithms [32].

### Outcomes

The primary outcome was the burden of patient-reported symptoms during the year after surgery, measured using the Edmonton Symptom Assessment System (ESAS) [33]. The ESAS is a validated symptom assessment tool assessing the severity of nine common cancer-associated symptoms (anxiety, depression, drowsiness, appetite, nausea, pain, dyspnea, tiredness, and wellbeing), and is a reliable measure of health-related quality of life [34–37]. Each symptoms is graded on a scale of 0 (no symptoms) to 10 (worst possible symptoms) and a sum off the scores is presented as an overall symptom distress score [35]. Moderate-to-severe symptoms are defined as scores ≥ 4 for individual symptoms and ≥ 40 for total scores [38]. A 1-point difference in individual scores and 10-point difference in overall scores between patient groups was considered clinically significant [39, 40]. The difference in individual symptom scores has previously been defined using anchor-based methods [39]. The total score was based on the global distress score, for which no global minimal clinical important difference has been agreed upon [41]. Based on previous evidence and the distribution based-approach, we used a 10-point difference to maximize specificity and minimize the possibility of falsely identifying a difference in symptom burden [40, 42]. In Ontario, the collection of ESAS scores is mandated at each RCC visit. By 2015 an estimated 61% of cancer patients in Ontario were screened with the ESAS [43].

One-year mortality was measured as a secondary outcome. All patients were followed until the earlier of one-year post-surgery or death.

### Covariates

Patients were characterized by age, sex, income quintile, geographic location (urban vs rural), burden of comorbid illnesses (high vs low), presence of frailty, and pre-operative ESAS scores. Income quintile was determined based on the median income of a patient’s neighborhood relative to incomes across Ontario [44]. Geographic location was dichotomized using the rurality index [45]. The Johns Hopkins Adjusted Clinical Groups® (ACG) System Version 10 was used to identify the burden of comorbid illness. Patients with ≥ 10 Aggregated Diagnosis Groups were classified as having a high burden of comorbid illnesses [46]. A patient was identified as frail if they had at least one diagnosis from 12 clusters of frailty-related conditions specified by the ACG system [47, 48]. Clinical characteristics included cancer site and stage, year of diagnosis, receipt of neo-adjuvant therapy, surgical procedure, length of stay (LOS) during the index admission, ICU LOS, duration of mechanical ventilation, and hospital disposition [49, 50]. Details of all covariates are available in Additional file 1: eTable 3.

### Statistical analysis

Baseline characteristics were assessed using descriptive statistics. Standardized differences were used to compare characteristics between patients who did or did not experience an unexpected ICU admission. Characteristics of the initial hospitalization and secondary outcomes were compared across patient groups using the Chi-square and Wilcoxon–Mann–Whitney tests.

The overall and monthly prevalence of moderate-to-severe symptoms during the year after surgery were compared across patient groups. We used a modified log-Poisson model with an autoregressive correlation structure to estimate the trajectory of a patients’ probability of having a moderate-to-severe symptom burden, while adjusting for confounders and accounting for repeated measurements within the same patient [51, 52]. The models were adjusted for age, sex, income, geographic location, comorbidity status, frailty, cancer site and stage, receipt of neo-adjuvant therapy, and year. Additionally, we hypothesized that the change in symptom burden over time was nonlinear and included a quadratic term for time. Finally, an interaction term between time and unexpected ICU admission evaluated the impact of an unexpected ICU admission on symptom trajectories. Unique models were built for total scores and each individual symptom score.

We then proceeded with a granular evaluation of the trends in ESAS scores across patient groups. This analysis was restricted to patients with two or more ESAS assessments during the year after surgery. If two ESAS assessments were recorded within the same month these scores were combined into a single assessment using the highest score for each symptom. Generalized linear mixed models with a spatial correlation structure and an unstructured covariance matrix were used to model symptom trajectories. These models were adjusted for the same characteristics as the modified log-Poisson analysis.

We performed two sensitivity analyses. First, we compared symptom trajectories across patients with an unexpected ICU admission stratified by the receipt of mechanical ventilation. Second, in patients who had both pre- and post-operative ESAS assessments we evaluated the impact of an unexpected ICU admission on a patient’s symptom trajectory relative to their pre-surgical status. The change in symptom scores was calculated as the post-operative ESAS score minus the pre-operative score. A negative score indicated a decrease, and a positive score an increase, in symptom burden. Multivariable models adjusted for the same confounders as the primary analysis were used for both sensitivity analyses.

Data were missing for location of residence and income in 0.1% and 0.2% of the cohort respectively. We used a complete-case analysis approach for the multivariable analysis. Stage at diagnosis was missing in 11.5% of patients. Because staging data may not be missing at random, a distinct “missing” category was used [53].

All analyses were performed using SAS software (version 9.4; SAS Institute Inc., Cary, North Carolina). Standardized differences > 0.10 and a two-tailed p-values < 0.05 were considered statistically significant [54].

## Results

Among the 31,664 older adults who survived high-intensity cancer surgery and had contact with a RCC, 16,560 (52.3%) completed at least one ESAS assessment during the year after surgery (Additional file 1: eFigure 1). The average age was 76.5 (± 5.0) years, 7,195 (43.4%) were female, and 11,620 (70.2%) had a gastrointestinal malignancy. Patients who completed an ESAS assessment were younger, diagnosed in a later year, and less often had a genitourinary malignancy than those who did not complete an assessment (Additional file 1: eTable 4).

Unexpected ICU admission occurred for 1,503 (9.1%) patients. The characteristics of patients by ICU group are presented in Table 1. Patients with an unexpected ICU admission had a longer median hospital LOS (13 vs 7 days; *p* < 0.001) and were less likely to be discharged home (87.8% vs 97.3%; *p* < 0.001). Among those with an unexpected ICU admission over two-thirds received mechanical ventilation (*n* = 1,050; 69.9%). Most ventilated patients received ventilation for seven or fewer days (*n* = 973; 92.7%).

**Table 1:** Baseline characteristics and initial hospital outcomes stratified by unexpected ICU admission status

	All patients (*n* = 16,560)	Unexpected ICU Admission		
		Yes (*n* = 1,503)	No (*n* = 15,057)	
*Baseline characteristics*				*Standardized difference* ^a^
Age, mean (± SD)	76.5 (5.0)	78.9 (5.0)	76.5 (4.9)	0.08
Age group, * n* (%)				
70–74	6,960 (42.0)	590 (39.3)	6,370 (42.3)	0.06
75–79	5,279 (31.9)	469 (31.2)	4,810 (31.9)	0.02
80–84	3,075 (18.6)	320 (21.3)	2,755 (18.3)	0.08
≥ 85	1,246 (7.5)	124 (8.3)	1,122 (7.5)	0.03
Female, * n* (%)	7,195 (43.4)	613 (40.8)	6,582 (43.7)	0.06
Income quintile, * n* (%)				
1–lowest	3,065 (18.5)	289 (19.2)	2,776 (18.4)	0.02
2	3,483 (21.0)	304 (20.2)	3,179 (21.1)	0.02
3	3,261 (19.7)	304 (20.2)	2,957 (19.6)	0.01
4	3,284 (19.8)	292 (19.4)	2,992 (19.9)	0.01
5–highest	3,435 (20.7)	311 (20.7)	3,124 (20.7)	< 0.01
Rural residence, * n* (%)	1,955 (11.8)	176 (11.7)	1,779 (11.8)	< 0.01
High comorbidity burden, * n* (%)	6,973 (42.1)	677 (45.0)	6,296 (41.8)	0.07
Frailty, * n* (%)	1,181 (7.1)	140 (9.3)	1,041 (6.9)	0.09
Cancer type, * n* (%)				
GI	11,620 (70.2)	1,115 (74.2)	10,505 (69.8)	0.10
GU	1,835 (11.1)	108 (7.2)	1,727 (11.5)	0.15
BP	3,105 (18.8)	280 (18.6)	2,825 (18.8)	< 0.01
Procedure				
Colectomy	9,408 (56.8)	743 (49.4)	8,665 (57.5)	0.16
Nephroureterectomy	1,743 (10.5)	100 (6.7)	1,643 (10.9)	0.15
Lobectomy	3,000 (18.1)	257 (17.1)	2,743 (18.2)	0.03
Other	2,409 (14.5)	403 (26.8)	2,006 (13.3)	0.34
Stage, * n* (%)				
1	2,432 (14.7)	174 (11.6)	2,258 (15.0)	0.10
2	4,568 (27.6)	429 (28.5)	4,139 (27.5)	0.02
3	5,670 (34.2)	481 (32.0)	5,189 (34.5)	0.05
4	1,992 (12.0)	192 (12.8)	1,800 (12.0)	0.02
Missing	1,898 (11.5)	227 (15.1)	1,671 (11.1)	0.12
Neo-adjuvant therapy, *n* (%)	1,487 (9.0)	145 (9.6)	1,342 (8.9)	0.03
Diagnosis in 2013 or later, * n* (%)	9,660 (58.3)	817 (54.3)	8,843 (58.7)	0.09
*Characteristics of the initial hospitalization*				*p-value*
ICU admission, * n* (%)	4,451 (26.9)	1,503 (100)	2,948 (19.6)	< 0.001
Mechanical ventilation, * n* (%)	1,050 (69.9)	1,050 (69.9)	–	–
Duration of mechanical ventilation (days), * n* (%)				
0	15,510 (93.7)	453 (30.1)	15,057 (100)	< 0.001
1–2	321 (21.4)	321 (21.4)	–	
3–7	652 (43.4)	652 (43.4)	–	
8–14	53 (3.5)	53 (3.5)	–	
> 14	24 (1.6)	24 (1.6)	–	
Hospital length of stay (days), median (IQR)	8 (5–11)	13 (9–21)	7 (5–10)	< 0.001
Hospital disposition, * n* (%)				
Home without homecare	9,448 (57.1)	782 (52.0)	8,911 (59.2)	< 0.001
Home with homecare	6,519 (39.4)	537 (35.7)	5,737 (38.1)	
Inpatient rehab	525 (3.2)	174 (11.6)	351 (2.3)	
Nursing home	62 (0.4)	7 (0.5)	55 (0.4)	

ICU, intensive care unit; SD, standard deviation; IQR, interquartile range; GI, gastrointestinal; GU, genitourinary; BP, bronchopulmonary

^a^standardized difference < 0.10 considered negligible difference[54]

More patients with an unexpected ICU admission died within a year of surgery compared to those without (18.7% vs 11.7%; *p* < 0.001). Fewer patients with an unexpected ICU admission received adjuvant therapy (29.9% vs 33.3%; *p* = 0.008).

### Burden of patient-reported symptoms

**Fig. 1:**
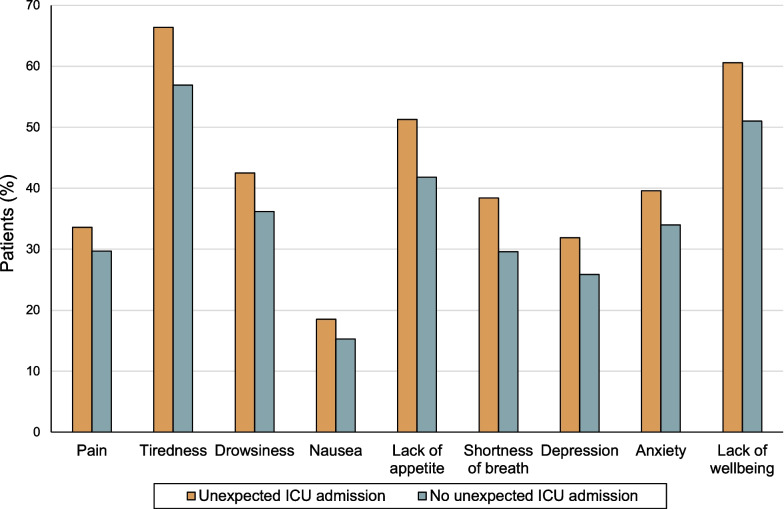
Proportion of patients who experienced moderate-to-severe symptoms at least once during the year after surgery, stratified by unexpected ICU admission status

A total of 77,411 ESAS assessments were analyzed, with a median of 3 (IQR 1–6) assessments per patient. There were no differences in the number of ESAS assessments between patients with and without an unexpected ICU admission (Additional file 1: eTable 5). During the year after surgery more patients with an unexpected ICU admission experienced moderate-to-severe symptoms (Additional file 1: eFigure 2). The most commonly reported moderate-to-severe symptoms were tiredness (57.8%), poor wellbeing (51.9%), and lack of appetite (42.6%), (Fig. 1).

**Fig. 2:**
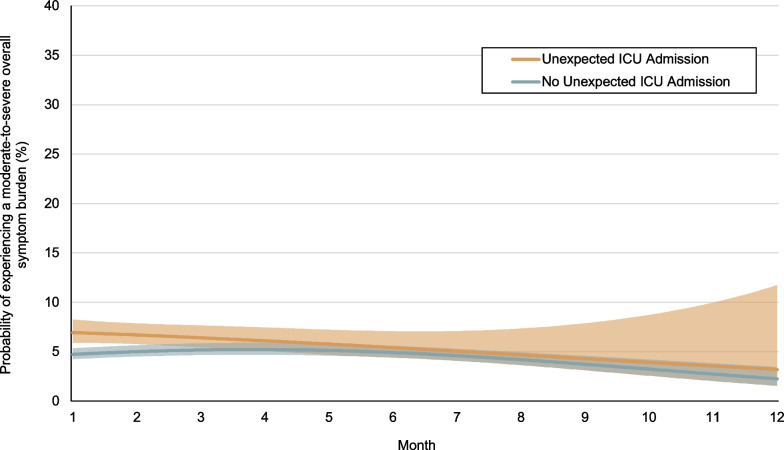
Trajectory of the probability of experiencing a moderate-to-severe overall symptom burden during the year after surgery, adjusted for baseline patient characteristics. * adjusted for age, sex, income quintile, geographic location, burden of comorbid illnesses, frailty, cancer site and stage, receipt of neo-adjuvant therapy, and year of diagnosis. † figure represents the average trajectories of the probability of experiencing a moderate-to-severe symptom burden for a male patient, age 70–74, with a low burden of comorbid illness, not identified as frail, diagnosed with stage one bronchopulmonary cancer in 2013, who did not receive neo-adjuvant treatment, and reside in an urban region in the lowest income quintile

Longitudinal analysis, adjusted for baseline characteristics demonstrated that the trends in the probability of experiencing a moderate-to-severe burden of total symptoms were different between patients with and without an unexpected ICU admission (*p* < 0.001, Fig. 2). Among patients with an unexpected ICU admission the average probability of experiencing a moderate-to-severe symptom burden decreased throughout the year after surgery, from 6.9% (95% CI 5.8–8.3%) during the first month, to 6.1% (95% CI 5.0–7.4%) at 120 days, and finally 3.2% (95% CI 0.9–11.7%) at the end of the year. Conversely, among patients without an unexpected ICU admission the average probability of experiencing moderate-to-severe symptoms initially increased from 4.7% (95% CI 4.2–5.3%) during the first month after surgery to 5.2% (95% CI 4.6–5.9%) at 120 days, before starting to decline, ending at 2.3% (95% CI 1.5–3.5%) at the end of the year. Overall, these trends demonstrated that the probability of experiencing a moderate-to-severe symptom burden was 1.6-fold greater among patients with an unexpected ICU admission compared to this without an unexpected admission (RR 1.64, 95% CI 1.31–2.05), (Additional file 1: eTable 6a).

The association between unexpected ICU admission and an increased probability of moderate-to-severe symptoms was demonstrated across all individual symptoms other than pain, with the greatest relative differences demonstrated among nausea, shortness of breath, and drowsiness (Additional file 1: eFigure 3).

### Changes in ESAS scores over time

**Fig. 3:**
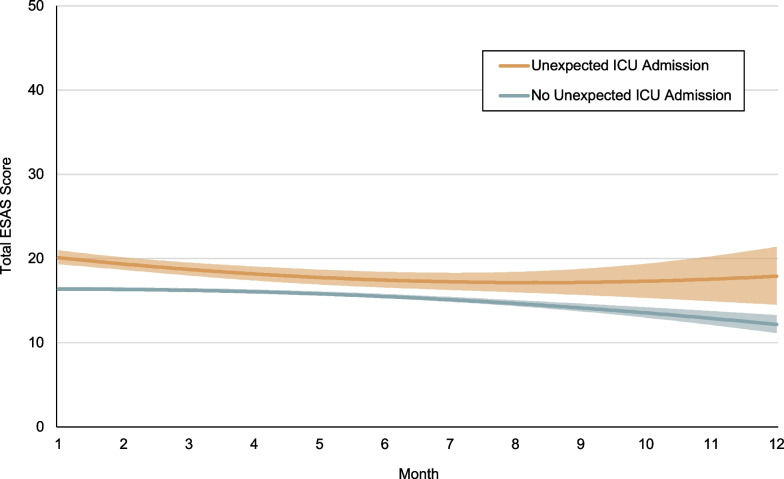
Trajectory of overall symptom burden during the year after surgery, adjusted for baseline patient characteristics. * adjusted for age, sex, income quintile, geographic location, burden of comorbid illnesses, frailty, cancer site and stage, receipt of neo-adjuvant therapy, and year of diagnosis. † figure represents average symptom trajectories for a male patient, age 70–74, with a low burden of comorbid illness, not identified as frail, diagnosed with stage one bronchopulmonary cancer in 2013, who did not receive neo-adjuvant treatment, and reside in an urban region in the lowest income quintile

The analysis of trends in ESAS scores was restricted to the 11,229 (67.8%) patients who completed ≥ 2 ESAS assessments and included 37,524 individual ESAS assessments (Additional file 1: eTable 7). After adjusting for baseline characteristics, the trajectories of patient-reported symptom burden (total ESAS scores) were statistically different between patients with and without an unexpected ICU admission (Fig. 3, Additional file 1: eTable 8a; *p* < 0.001). In patients without an unexpected ICU admission, total ESAS scores consistently decreased from an average score of 16.4 (95% CI 16.1–16.6) during the first month after surgery to 12.2 (95% CI 11.1–13.3) during the last month. In patients with an unexpected ICU admission, total ESAS scores initially decreased from an average of 20.1 (95% CI 19.2–21.0) during the first month to 17.2 (95% CI 15.9–18.4) eight months after surgery. However, by nine months total ESAS scores started to increase and eventually reached an average of 17.9 (95% CI 14.4–21.4) in the last month. At no point during the year after surgery did the difference in total ESAS scores between groups reach the threshold for clinical significance (10 points).

When analyzing individual symptoms, the only symptom with a clinically significant change in burden between ICU groups was tiredness (Additional file 1: eFigure 4). Across all other symptoms the differences in patient-reported scores did not exceed one point either within or between groups.

### Impact of mechanical ventilation on symptom burden

After adjusting for baseline characteristics, the trends in the probability of experiencing a moderate-to-severe overall symptom burden differed significantly between the 1,050 patients who received mechanical ventilation and the 453 patients who experienced an unexpected ICU admission without mechanical ventilation (*p* < 0.001). Specifically, among patients who received mechanical ventilation there was a trend toward a decreasing probability of experiencing a moderate-to-severe symptom burden during the year after surgery, whereas among ICU patients who did not receive ventilation there was no change in their likelihood of experiencing moderate-to-severe symptoms during the year. Despite the slight differences in trends, throughout the year there was no difference in the likelihood of experiencing a moderate-to-severe symptoms between ICU patients who did or did not receive mechanical ventilation (RR 1.02, 95% CI 0.67–1.57), (Additional file 1: eTable 6b). Furthermore, among the 984 patients who experienced an unexpected ICU admission and completed ≥ 2 ESAS assessments, mechanical ventilation did not impact total ESAS scores (difference in total scores: 0.56 points, 95% CI -2.11–3.23), or the trajectory of these scores during the year (*p* = 0.99), (Additional file 1: eTable 8b).

### Impact of pre-operative symptom burden

**Fig. 4:**
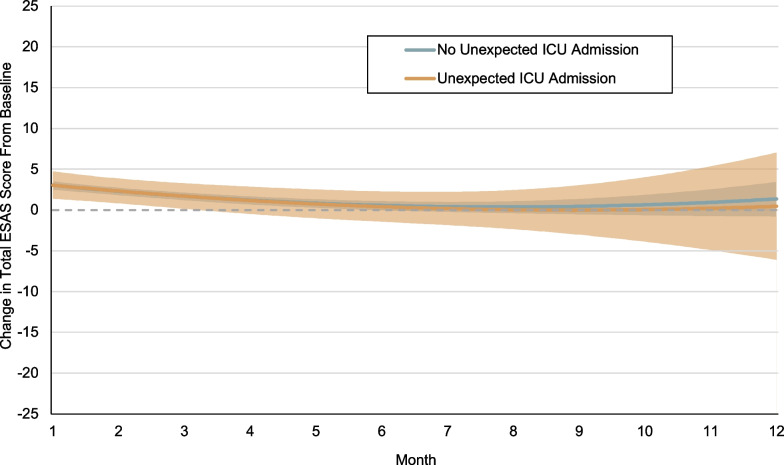
Change in overall symptom burden during the year after surgery relative to pre-operative symptom burden, adjusted for baseline patient characteristics.* adjusted for age, sex, income quintile, geographic location, burden of comorbid illnesses, frailty, cancer site and stage, receipt of neo-adjuvant therapy, and year of diagnosis.† figure represents the average change in symptoms for a male patient, age 70–74, with a low burden of comorbid illness, not identified as frail, diagnosed with stage one bronchopulmonary cancer in 2013, who did not receive neo-adjuvant treatment, and reside in an urban region in the lowest income quintile

A total of 3,720 (22.5%) patients had both pre- and post-operative ESAS scores. Patients with unexpected ICU admissions reported a higher overall symptom distress score prior to surgery (Additional file 1: eTable 9). Consistent with the primary analysis, there was no statistically or clinically significant difference in the trajectory of the change in total ESAS scores relative to baseline between patient groups (Fig. 4, Additional file 1: eTable 8c). These findings were consistent for individual symptoms (Additional file 1: eFigure 5).

## Discussion

In this population-based study, we found that older adults who had an unexpected ICU admission following cancer surgery were 1.6-times more likely to experience a moderate-to-severe burden of cancer symptoms during the subsequent year relative to those who did not have an unexpected ICU admission. Despite this increased risk, the probability that the average patient who survived an unexpected ICU would describe their overall symptom burden as moderate-to-severe was less than 10% throughout the year. Furthermore, granular analysis of ESAS scores demonstrates that after adjusting for baseline characteristics, an unexpected ICU admission was not associated with a clinically relevant difference in ESAS scores throughout the year after surgery. Our findings suggest that while post-operative critical illness may be associated with a minor increase in the risk of a worse functional outcome among older adults who survive their hospitalization after high-intensity cancer surgery, most patients do not experience a high burden of negative symptoms. This evaluation provides unique insights into long-term outcomes among older adults who require ICU care and highlights the importance of understanding the patient experience when making treatment decisions.

Our work appears to challenge previous studies which suggest that older adults admitted to the ICU experience a high rate of functional decline [9, 13–15]. These findings may relate to differences between our study population and the previous literature. In focusing on individuals deemed fit for a major surgical procedure, our population likely had a higher baseline functional status than the average older adult admitted to ICU. Patients in this study may therefore have been less vulnerable to functional decline [55]. It is also plausible that the indication for ICU admission among surgical patients is related to a reversible post-operative event. Consequently, they may have a greater likelihood of clinical and functional recovery.

In addition to focusing on patients undergoing cancer surgery, we approached the assessment of functional outcomes in a unique manner. Rather than focusing on objective measures of function as previously done, our study focused on patient-reported functional status [15, 56]. Focusing on patient reports is essential as individuals evaluate their symptoms in comparison to their own experience. Prior data suggest that relative to the general population, patients who survive a cancer diagnosis often develop increased resiliency and sense of meaning within their life [57, 58]. Likewise, having experienced severe illness, patients with cancer may have developed benefit-finding techniques; skills which allow them to find positive meaning during recovery [59, 60]. It is plausible that patients included in our study were more likely to rate their outcomes favorably compared to patients previously included in the literature.

Our study is not the first to demonstrate that critical illness is not associated with an increased burden of symptoms among older adults. Examining 400 mechanically ventilated patients, Hamilton et al*.* demonstrated that older age was associated with a decreased risk of developing depressive symptoms after critical illness [61]. The authors speculated that the protective effect of older age may be related to decreased societal demands on older individuals or an expectation of physical limitations later in life. Similarly, our results suggest that while critical illness may be associated with an initial increase in moderate-to-severe symptoms, over time the symptom burden decreases. It is unclear if this trajectory represents a decrease in functional limitations or patients adapting to changes in their functional status. Regardless, our results demonstrate that from the patient’s perspective the symptom burden during the year after surgery is not significantly altered by an ICU admission.

Our results do not suggest that all older adults admitted to the ICU after cancer surgery will have outcomes equivalent to those who did not experience critical illness. The results of this study cannot be used in isolation and should be combined with all the data available to clinicians when discussing transfer to ICU and implementation of aggressive life-prolonging measures. Indeed, one in five older adults admitted to the ICU after cancer surgery will die in hospital with key patient groups, such as those with baseline frailty being at higher risk for poor outcomes [8, 62]. However, the ability to accurately determine which patients will survive their hospitalization at the time of ICU admission remains challenging and there may be discrepancies in perceived outcomes between the surgical and ICU teams, patients, and families [63–65]. Given these differences in perspective and clinical uncertainty, our results support the use of time-limited trials of ICU therapies [66, 67]. These time-limited trials can provide the clinical team with additional information to facilitate prognostication, improve communication, and ensure treatment plan remains patient-centered [68, 69]. It is also important to note that nine months after surgery, symptoms scores among patients who experienced an unexpected ICU admission started to increase. Previous evidence demonstrates that the median time alive and at home among this patient population is about 16 months [8]. Although we demonstrated only a small change in symptom trajectory, this change may indicate a patient is nearing the end of their life. Furthermore, this finding reinforces that while older adults can have a reasonable quality of life after an ICU admission, these admissions still represent high-risk events and have significant implications regarding long-term survival.

Our results must be interpreted in the context of specific limitations. Although the ESAS is routine across all RCCs, the performance of ESAS assessments at additional hospital sites is voluntary [43]. This voluntary nature limits the applicability of our results among populations less likely to complete ESAS, including marginalized individuals and those who identify as recent immigrants [70]. It is therefore important to understand the unique patient experience when discussing treatment options and outcomes. Our assessment is also limited by survival bias, as patients who survived longer had a greater opportunity to complete ESAS assessments. However, previous data demonstrate that symptom burden increase prior to death [71]. Consequently, the differential death rates in our study should bias our results to finding an increase in symptom burden among patients with unexpected ICU admission and a larger difference in symptom trajectories. While there may be concern that more patients with an unexpected ICU admission were too sick to attend outpatient follow-up and complete an ESAS assessment, we demonstrated that the number of completed assessments was the same between patient groups. Finally, it is possible that our results are subject to misclassification bias. It is possible that some of the patients with an expected ICU admission, experienced a complication that would have resulted in an unexpected ICU admission had they not been admitted to the ICU immediately after surgery. Misclassification would bias our estimates of differences in symptom trajectories toward the null. It is unlikely that misclassification bias had major impact on our results as we classified all patients who required mechanical ventilation as having experienced an unexpected ICU admission regardless of what day they were admitted to the ICU.

In summary, our study demonstrates that after critical illness older adults who have recently undergone major cancer surgery are able to return to a similarly perceived quality of life as those who did not require ICU intervention. Our data suggest that neither the presence of a malignancy nor a specific age cut-off should be a barrier to ICU admission. These results highlight the importance of understanding the impact of the proposed treatments on patient-reported outcomes and reaffirm the role of life-prolonging measures among older adults.

## Supplementary Information

**Additional file 1.** Supplementary Tables and Figures.

## Data Availability

The dataset from this study is held securely in coded form at ICES. While legal data sharing agreements between ICES and data providers (e.g., healthcare organizations and government) prohibit ICES from making the dataset publicly available, access may be granted to those who meet pre-specified criteria for confidential access, available at www.ices.on.ca/DAS (email: das@ices.on.ca).

## Abbreviations

ACG: Adjusted Clinical Groups
BP: Bronchopulmonary
CI: Confidence interval
ESAS: Edmonton Symptom Assessment System
GI: Gastrointestinal
GU: Genitourinary
LOS: Length of stay
ICU: Intensive care unit
IQR: Interquartile range
RCC: Regional cancer center
SD: Standard deviation

## Code availability

The codes used for this study is available upon reasonable request made to the corresponding author.
