# Supplementary material for: The impact of unexpected intensive care unit admission after cancer surgery on long-term symptom burden among older adults: a population-based longitudinal analysis
Source: Crit Care. 2023 Apr 25;27:162. doi: 10.1186/s13054-023-04415-8 (PMC10127328; doi:10.1186/s13054-023-04415-8)
Supplement: Supplementary file 1 — Additional file 1. Supplementary Tables and Figures. [file 13054_2023_4415_MOESM1_ESM.docx]

**Supplementary Online Content**

**eTable 1. Details of the administrative datasets**

**eTable 2. Diagnostic and procedure codes utilized for patient identification**

**eTable 3. Definition of covariates**

**eTable 4. Baseline characteristics stratified by ESAS assessment status**

**eTable 5. Details of ESAS assessments**

**eTable 6. Modified log-Poisson analyses**

**eTable 7. Baseline characteristics with two or more ESAS assessments, stratified by unexpected ICU status**

**eTable 8. Adjusted general linear mixed models**

**eTable 9. Pre-operative symptom burden**

**eFigure 1. Cohort creation**

**eFigure 2. Unadjusted trends in the proportion of patients experiencing moderate-to-severe symptoms**

**eFigure 3. Trajectory of the probability of experiencing a moderate-to-severe burden of individual symptoms during the year after surgery, adjusted for baseline patient characteristics**

**eFigure 4. Trajectory of individual symptom burden during the year after surgery, adjusted for baseline patient characteristics**

**eFigure 5. Sensitivity analysis examining the trajectory of the change in individual symptom burden during the year after surgery relative to pre-operative status**

This supplementary material has been provided by the authors to give readers additional information about their work.

**eTable 1. Details of the administrative databases**

Data were obtained from several administrative databases in Ontario, Canada. All databases are held at ICES

| **Database** | **Description** |
| --- | --- |
| **Ontario Cancer Registry (OCR)** | The OCR is a passive, provincial registry all incident cancer diagnoses in Ontario. It includes 96% of cancer diagnoses in the province. Information included in the registry: cancer topography and morphology/histology, details on diagnosis (e.g., types of contributing information to the diagnosis, dates). |
| **Registered Patient Database (RPDB)** | The RPDB is an ICES database derived from all administrative data sources and provides demographic data including age, patient residence, vital status, date of last contact with the healthcare system, and OHIP eligibility. |
| **Canadian Institute of Health Information Discharge Abstract Database (CIHI-DAD)** | The DAD captures administrative, clinical, and demographic information on all hospital discharges (including deaths, sign-outs and transfers). |
| **National Ambulatory Care Reporting System (NACRS)** | The NACRS collects data elements describing emergency health services provided by emergency departments in Ontario, including diagnoses, procedures, and administrative information such as wait times. |
| **Ontario Health Insurance Plan (OHIP)** | The OHIP database contains all physician billing data including information on diagnoses as well as services provided, such as receipt of surgery, chemotherapy, and radiotherapy. |
| **Continued Care Reporting System (CCRS)** | The CCRS contains information on individuals receiving facility based continuing care, including medical long-term care, rehabilitation, geriatric assessment, respite care, palliative care, and nursing home care. |
| **Edmonton Symptom Assessment System (ESAS)** | The ESAS contains patient-reported symptom assessments for nine common cancer-associated symptoms, anxiety, depression, drowsiness, appetite, nausea, pain, dyspnea, tiredness, and wellbeing |

**eTable 2. Diagnostic and procedure codes utilized for patient identification**

| **Cancer site** | **Sub-type** | **ICD.O-3 code** | **Canadian Classification of Health Intervention Procedure Code** |
| --- | --- | --- | --- |
| **Gastrointestinal** | **Esophageal** | C15.0 to C15.9 | Esophagectomy: 1NA87^^, 1NA88^^, 1NA89^^, 1NA90^^, 1NA91^^, 1NA92^^  *Excluding: 1NA87BA^^, 1NA87DA^^, 1NA87LA^^* |
|  | **Gastric** | C16.0 to C16.9 | Gastrectomy: 1NF87^^, 1NF89^^, 1NF90^^, 1NF91^^, 1NF92^^  *Excluding:* 1NF87BA^^, 1NF87DA^^, 1NF87LA^^ |
|  | **Hepato-pancreatico-biliary** | Liver C22.0, C22.1  Biliary C23.9, C24.0, C24.1, C24.8, C24.9  Pancreas C25.0 to C25.9 | Hepatectomy: 1OA87^^  Bile ducts: 1OE87^^, 1OE89^^  *excluding 1OE87BA^^, 1OE89BA^^*  Pancreatectomy: 1OJ87^^ (distal), 1OJ89^^ (distal), 1OK87^^ (whipple), 1OK89^^ (whipple), 1OK91^^ (whipple) |
|  | **Colo-rectal and enteric** | Small bowel C17.0 to C17.9  Colon C18.0 to C18.9  Rectum C19.9, C20.9 | Colectomy and enterectomy: 1NK87^^, 1NM87^^, 1NM89^^, 1NM91^^, 1NQ87^^, 1NQ89^^ |
| **Genitourinary**  **(excluding penis, seminal vesicle, urethra)** | **Kidney and ureter** | C64.9, C65.9, C66.9 | Nephroureterectomy: 1PC87^^, 1PC89^^, 1PC91^^, 1PE87^^, 1PE89^^, 1PE91^^  *Excluding 1PG87BA^^, 1PG89BA^^* |
|  | **Bladder** | C67.0 to C67.9 | Radical cystectomy: 1PL87^^, 1PM87^^, 1PM89^^, 1PM90^^, 1PM91^^, 1PM92^^,  *Excluding 1PL87BA^^, 1PL89BA^^, 1PM87BA^^, 1PM89BA^^* |
|  | **Prostate** | C61.9 | Prostatectomy: 1QT87^^, 1QT91^^  *Excluding 1QT87BA^^* |
|  | **Adrenal** | C74.0, C74.1, C74.9 | Adrenalectomy: 1PB87^^, 1PB89^^  *Excluding 1PB87DA^^, 1PB89DA^^* |
| **Bronchopulmonary** | | C30.0 to 34.9, C38.1 to 39.9 | Lobectomy: 1GR.87^^, 1GR89^^, 1GR91^^, 1GT87^^  Pneumonectomy: 1GT89^^, 1GT91^^ |

ICD = International Classification of Diseases 10^th^ Edition

**eTable 3. Definition of covariates**

| **Variable** | **Definition** | **Source** | **Type** | **Analysis format** |
| --- | --- | --- | --- | --- |
| *Age* | Age at diagnosis | Ontario Cancer Registry | Categorical | 70-74  75-79  80-84  >85 |
| *Sex* | Sex | Ontario Cancer Registry | Dichotomous | Male/Female |
| *Income quintile* | Ecologic measure of income based on the median income of a patient’s postal code of residence (1) | Canadian Census  Registered Persons Database | Categorical | 1^st^ (lowest)  2^nd^  3^rd^  4^th^  5^th^ (highest) |
| *Rural resident* | Determined with postal code of residence as per national census definition of a community <10,000 people (2) | Registered Persons Database | Dichotomous | Urban/Rural |
| *Comorbidity burden* | Measured using the Johns Hopkins Adjusted Clinical Groups system score based on health services use with a 24-month look-back window prior to the date of cancer diagnosis whereby the 32 aggregated diagnosis groups (ADG) are summed to create a total score (3) | CIHI-Discharge abstract database  Ontario Health Insurance Plan | Dichotomous | <10  >10 |
| *Frailty score* | Measured using the Johns Hopkins Aggregates Clinical Diagnoses frailty marker (4) | CIHI-Discharge abstract database  National Ambulatory Care Reporting System | Dichotomous | Frailty  No frailty |
| *Cancer type* | Type of primary cancer based on ICD-O.3 codes (eTable 2) | Ontario Cancer Registry | Categorical | Gastrointestinal  Genitourinary  Bronchopulmonary |
| *Cancer stage* | TMN stage at time of diagnosis (5) | Ontario Cancer Registry | Categorical | 1  2  3  4  Missing |
| *Neo-adjuvant therapy* | Receipt of chemotherapy or chemoradiation therapy within 180 days prior to surgery (6, 7) | Ontario Cancer Registry | Dichotomous | Yes/no |
| *Year of diagnosis* | Year of cancer diagnosis date | Ontario Cancer Registry | Continuous | One-year change, centered to 2013 |
| *Length of hospital stay* | Duration of index admission, measured in days | CIHI-Discharge abstract database | Continuous | One-day change |
| *Length of ICU stay* | Total duration of ICU stay during the index admission, measured in days | CIHI-Discharge abstract database | Continuous | One-day change |
| *Duration of mechanical ventilation* | Longest continuous duration of mechanical ventilation during the index admission, measured in days | CIHI-Discharge abstract database | Continuous | One-day change |
| *Hospital disposition* | Discharge disposition at completion of index admission | CIHI-Discharge abstract database | Categorical | Home without homecare  Home with homecare  Inpatient rehab  Nursing home |

ICU = intensive care unit

1. Wilkins R. Use of postal codes and addresses in the analysis of health data. *Health Rep*. 1993;5(2):157-77
2. Kralj B. Measuring 'rurality' for purposes of health-care planning: an empirical measure for Ontario. *Ontario Medical Review*. 2000;October
3. Reid RJ, MacWilliam L, Verhulst L, Roos N, Atkinson M. Performance of the ACG case-mix system in two Canadian provinces. *Med Care*. Jan 2001;39(1):86-99.
4. Ho MM, Camacho X, Gruneir A, Bronskill SE. *Overview of Cohorts, In: Health System Use by Frail Ontario Seniors: An In-Depth Examination of Four Vulnerable Cohorts*. Institute for Clinical Evaluative Sciences; 2011.
5. Edge SB, Compton CC. The American Joint Committee on Cancer: the 7th edition of the AJCC cancer staging manual and the future of TNM. *Ann Surg Oncol*. Jun 2010;17(6):1471-4. doi:10.1245/s10434-010-0985-4
6. Nam RK, Cheung P, Herschorn S, et al. Incidence of complications other than urinary incontinence or erectile dysfunction after radical prostatectomy or radiotherapy for prostate cancer: a population-based cohort study. *Lancet Oncol*. Feb 2014;15(2):223-31. doi:10.1016/S1470-2045(13)70606-5
7. Kagedan DJ, Abraham L, Goyert N, et al. Beyond the dollar: Influence of sociodemographic marginalization on surgical resection, adjuvant therapy, and survival in patients with pancreatic cancer. *Cancer*. Oct 2016;122(20):3175-3182. doi:10.1002/cncr.30148

**eTable 4. Baseline characteristics stratified by ESAS assessment status**

|  | Post-operative ESAS assessment | | Standardized difference |
| --- | --- | --- | --- |
|  | Yes  (n=16,560) | No  (n=15,194) |  |
| Age, mean (±SD) | 76.5 ± 5.0 | 77.3 ± 5.3 | 0.15 |
| Age group, n (%) |  |  |  |
| 70 – 74 | 6,960 (42.0) | 5,626 (37.0) | 0.10 |
| 75 – 79 | 5,279 (31.9) | 4,673 (30.8) | 0.02 |
| 80 – 84 | 3,075 (18.6) | 3,226 (21.2) | 0.07 |
| ≥ 85 | 1,246 (7.5) | 1,669 (11.0) | 0.12 |
| Female, n (%) | 7,195 (43.4) | 6,443 (42.4) | 0.02 |
| Income quintile, n (%) |  |  |  |
| 1 – lowest | 3,065 (18.5) | 3,026 (19.9) | 0.04 |
| 2 | 3,483 (21.0) | 3,311 (21.8) | 0.02 |
| 3 | 3,261 (19.7) | 2,940 (19.3) | 0.01 |
| 4 | 3,284 (19.8) | 2,905 (19.1) | 0.02 |
| 5 – highest | 3,435 (20.7) | 2,978 (19.6) | 0.03 |
| Rural residence, n (%) | 1,955 (11.8) | 1,619 (10.7) | 0.04 |
| High comorbidity burden, n (%) | 6,973 (42.1) | 6,815 (44.9) | 0.06 |
| Frailty, n (%) | 1,181 (7.1) | 1,255 (8.3) | 0.04 |
| Cancer type, n (%) |  |  |  |
| GI | 11,620 (70.2) | 10,121 (66.6) | 0.08 |
| GU | 1,835 (11.1) | 2,719 (17.9) | 0.19 |
| BP | 3,105 (18.8) | 2,354 (15.5) | 0.09 |
| Procedure |  |  |  |
| Colectomy | 9,408 (56.8) | 8,908 (58.6) | 0.04 |
| Nephroureterectomy | 1,743 (10.5) | 2,673 (17.6) | 0.20 |
| Lobectomy | 3,000 (18.1) | 2,287 (15.1) | 0.08 |
| Other | 2,049 (12.4) | 1,326 (8.7) | 0.12 |
| Stage, n (%) |  |  |  |
| 1 | 2,432 (14.7) | 3,368 (22.2) | 0.19 |
| 2 | 4,568 (27.6) | 4,499 (29.6) | 0.04 |
| 3 | 5,670 (34.2) | 3,893 (25.6) | 0.19 |
| 4 | 1,992 (12.0) | 1,034 (6.8) | 0.18 |
| Missing | 1,898 (11.5) | 2,400 (15.8) | 0.13 |
| Neo-adjuvant therapy, n (%) | 1,487 (9.0) | 741 (4.9) | 0.16 |
| Diagnosis in 2013 or later, n (%) | 9,660 (58.3) | 6,218 (40.9) | 0.35 |

ICU = intensive care unit; SD = standard deviation; IQR = interquartile range; GI = gastrointestinal; GU = genitourinary; BP = bronchopulmonary

**eTable 5. Details of ESAS assessments**

|  | Unexpected ICU Admission | | Standardized difference |
| --- | --- | --- | --- |
|  | Yes | No |  |
| No. of ESAS assessments completed in the year after surgery, median (IQR) | 3 (1 – 6) | 3 (1 – 6) | 0.06 |
| No. of ESAS assessments completed in the year after surgery, n (%) |  |  |  |
| 1 | 475 (31.6) | 4,405 (29.3) | 0.05 |
| 2 – 5 | 635 (42.2) | 6,249 (41.5) | 0.02 |
| ≥ 6 | 393 (26.1) | 4,403 (29.2) | 0.07 |
| Days from surgery to first ESAS assessment, median (IQR) | 60 (38 – 110) | 51 (34 – 98) | 0.17 |
| Days from surgery to last ESAS assessment, median (IQR) | 225 (103 – 315) | 229 (105 – 313) | <0.01 |
| Days between first to last ESAS assessment, median (IQR) * | 98 (0 – 227) | 118 (0 – 231) | 0.06 |
| Pre-op and post-op ESAS assessment, n (%) | 375 (25.0) | 3,345 (22.2) | 0.06 |
| ESAS assessment completed, n (%) |  |  |  |
| Month 1 | 220 (14.6) | 3,068 (20.4) | 0.15 |
| Month 2 | 646 (43.0) | 7,050 (46.8) | 0.08 |
| Month 3 | 564 (37.5) | 5,839 (38.8) | 0.03 |
| Month 4 | 502 (33.4) | 5,282 (35.1) | 0.04 |
| Month 5 | 451 (30.0) | 4,934 (32.8) | 0.06 |
| Month 6 | 446 (29.7) | 4,439 (29.5) | <0.01 |
| Month 7 | 370 (24.6) | 4,076 (27.1) | 0.06 |
| Month 8 | 371 (24.7) | 3,666 (24.3) | 0.01 |
| Month 9 | 307 (20.4) | 3,080 (20.5) | <0.01 |
| Month 10 | 295 (19.6) | 2,641 (17.5) | 0.05 |
| Month 11 | 274 (18.3) | 2,552 (16.9) | 0.03 |
| Month 12 | 282 (18.8) | 2,729 (18.1) | 0.02 |

* restricted to those with 2 or more ESAS assessments

**eTable 6. Modified log-Poisson analyses**

**A: Estimating the trajectory of the probability of experiencing a moderate-to-severe overall symptom burden**

|  | Adjusted relative risk (95% CI) | p-value |
| --- | --- | --- |
| Time (change per month) | 1.10 (1.05 - 1.16) | <0.001 |
| Quadratic function of time (change per month^2^) | 0.99 (0.98 - 0.99) | <0.001 |
| Unexpected ICU admission | 1.64 (1.31 - 2.05) | <0.001 |
| Interaction between time and unexpected ICU admission | 0.89 (0.77 - 1.02) | 0.09 |
| Interaction between quadratic function of time and unexpected ICU admission | 1.01 (0.99 - 1.03) | 0.33 |
| Age group |  |  |
| 70 – 74 | Ref | - |
| 75 – 79 | 1.05 (0.97 - 1.15) | 0.21 |
| 80 – 84 | 0.98 (0.89 - 1.10) | 0.78 |
| ≥ 85 | 0.90 (0.76 - 1.06) | 0.19 |
| Female | 1.41 (1.31 - 1.52) | <0.001 |
| Income quintile |  |  |
| 1 – lowest | Ref | - |
| 2 | 1.01 (0.90 - 1.14) | 0.83 |
| 3 | 0.97 (0.86 - 1.09) | 0.61 |
| 4 | 0.95 (0.85 - 1.07) | 0.42 |
| 5 – highest | 0.96 (0.85 - 1.08) | 0.49 |
| Rural residence | 0.71 (0.62 - 0.81) | <0.001 |
| High comorbidity burden | 1.47 (1.37 - 1.59) | <0.001 |
| Frailty | 1.18 (1.04 - 1.35) | 0.01 |
| Cancer type |  |  |
| GI | 0.55 (0.50 - 0.60) | <0.001 |
| GU | 0.73 (0.64 - 0.84) | <0.001 |
| BP | Ref | - |
| Stage |  |  |
| 1 | Ref | - |
| 2 | 1.14 (1.00 - 1.30) | 0.06 |
| 3 | 1.09 (0.95 - 1.24) | 0.22 |
| 4 | 1.61 (1.40 - 1.86) | <0.001 |
| Missing | 1.54 (1.32 - 1.79) | <0.001 |
| Neo-adjuvant therapy | 1.05 (0.88 - 1.26) | 0.61 |
| Change in one year | 0.98 (0.97 - 0.99) | <0.001 |

p-value for differences in trajectories between patients with and without an unexpected ICU admission: p<0.001

**B: Sensitivity analysis estimating the impact of mechanical ventilation on the trajectory of the probability of experiencing a moderate-to-severe overall symptom burden among patients with an unexpected ICU admission**

|  | Adjusted relative risk (95% CI) | p-value |
| --- | --- | --- |
| Time (change per month) | 1.03 (0.87 – 1.23) | 0.71 |
| Quadratic function of time (change per month^2^) | 1.00 (0.98 – 1.02) | 0.80 |
| Receipt of mechanical ventilation | 1.02 (0.67 – 1.57) | 0.92 |
| Interaction between time and receipt of mechanical ventilation | 0.94 (0.72 – 1.22) | 0.63 |
| Interaction between quadratic function of time and receipt of mechanical ventilation | 0.99 (0.96 – 1.03) | 0.68 |
| Age group |  |  |
| 70 – 74 | Ref | - |
| 75 – 79 | 0.88 (0.69 – 1.14) | 0.34 |
| 80 – 84 | 1.04 (0.77 – 1.41) | 0.77 |
| ≥ 85 | 0.83 (0.52 – 1.31) | 0.42 |
| Female | 1.43 (1.15 – 1.79) | 0.002 |
| Income quintile |  |  |
| 1 – lowest | Ref | - |
| 2 | 1.01 (0.70 – 1.45) | 0.96 |
| 3 | 1.19 (0.86 – 1.64) | 0.30 |
| 4 | 1.01 (0.72 – 1.42) | 0.96 |
| 5 – highest | 1.37 (0.98 – 1.89) | 0.06 |
| Rural residence | 0.59 (0.39 – 0.90) | 0.01 |
| High comorbidity burden | 1.31 (1.05 – 1.65) | 0.02 |
| Frailty | 0.99 (0.70 – 1.41) | 0.96 |
| Cancer type |  |  |
| GI | 0.64 (0.48 – 0.85) | 0.002 |
| GU | 1.03 (0.69 – 1.52) | 0.90 |
| BP | Ref | - |
| Stage |  |  |
| 1 | Ref | - |
| 2 | 1.11 (0.74 – 1.65) | 0.62 |
| 3 | 1.20 (0.79 – 1.83) | 0.38 |
| 4 | 1.19 (0.76 – 1.86) | 0.44 |
| Missing | 1.40 (0.91 – 2.16) | 0.13 |
| Neo-adjuvant therapy | 1.00 (0.62 – 1.62) | 0.99 |
| Change in one year | 0.98 (0.95 – 1.02) | 0.34 |

p-value for differences in trajectories between ICU patients with and without mechanical ventilation: p<0.001

**eTable 7. Baseline characteristics of patients with two or more ESAS assessments, stratified by unexcepted ICU status**

|  | Unexpected ICU Admission | | Standardized difference |
| --- | --- | --- | --- |
|  | Yes  (n=984) | No  (n=10,245) |  |
| Age, mean (±SD) | 76.3 ± 4.70 | 76.1 ± 4.70 | 0.05 |
| Age group, n (%) |  |  |  |
| 70 – 74 | 416 (42.3) | 4,579 (44.7) | 0.05 |
| 75 – 79 | 320 (32.5) | 3,356 (32.8) | 0.01 |
| 80 – 84 | 191 (19.4) | 1,710 (16.7) | 0.07 |
| ≥ 85 | 57 (5.8) | 600 (5.9) | <0.01 |
| Female, n (%) | 382 (38.8) | 4,504 (44.0) | 0.10 |
| Income quintile, n (%) |  |  |  |
| 1 – lowest | 182 (18.5) | 1,831 (17.9) | 0.02 |
| 2 | 202 (20.5) | 2,165 (21.1) | 0.01 |
| 3 | 193 (19.6) | 2,036 (19.9) | 0.01 |
| 4 | 192 (19.5) | 2,085 (20.4) | 0.02 |
| 5 – highest | 215 (21.8) | 2,128 (20.8) | 0.03 |
| Rural residence, n (%) | 110 (11.2) | 1,135 (11.1) | <0.01 |
| High comorbidity burden, n (%) | 431 (43.8) | 4,206 (41.1) | 0.06 |
| Frailty, n (%) | 89 (9.0) | 635 (6.2) | 0.11 |
| Cancer type, n (%) |  |  |  |
| GI | 728 (74.0) | 7,197 (70.2) | 0.08 |
| GU | 69 (7.0) | 1,121 (10.9) | 0.14 |
| BP | 187 (19.0) | 1,927 (18.8) | <0.01 |
| Procedure |  |  |  |
| Colectomy | 454 (46.1) | 5,835 (57.0) | 0.22 |
| Nephroureterectomy | 64 (6.5) | 1,063 (10.4) | 0.14 |
| Lobectomy | 170 (17.3) | 1,860 (18.2) | 0.02 |
| Other | 296 (38.1) | 1,487 (14.5) | 0.38 |
| Stage, n (%) |  |  |  |
| 1 | 83 (8.4) | 1,231 (12.0) | 0.12 |
| 2 | 276 (28.0) | 2,659 (26.0) | 0.05 |
| 3 | 333 (33.8) | 3,907 (38.1) | 0.09 |
| 4 | 139 (14.1) | 1,380 (13.5) | 0.02 |
| Missing | 153 (15.5) | 1,068 (10.4) | 0.15 |
| Neo-adjuvant therapy, n (%) | 115 (11.7) | 1,024 (10.0) | 0.05 |
| Diagnosis in 2013 or later, n (%) | 525 (53.4) | 6,042 (59.0) | 0.11 |

ICU = intensive care unit; SD = standard deviation; IQR = interquartile range; GI = gastrointestinal; GU = genitourinary; BP = bronchopulmonary

**eTable 8. Adjusted general linear mixed models**

**A: Estimating the trajectory of overall symptom distress scores**

|  | Adjusted Beta coefficient (95% CI) | p-value |
| --- | --- | --- |
| Time (change per month) | 0.07 (-0.10 – 0.23) | 0.43 |
| Quadratic function of time (change per month^2^) | -0.03 (-0.05 – -0.02) | <0.001 |
| Unexpected ICU admission | 4.64 (3.42 – 5.86) | <0.001 |
| Interaction between time and unexpected ICU admission | -0.99 (-1.54 – -0.43) | <0.001 |
| Interaction between quadratic function of time and unexpected ICU admission | 0.09 (0.03 – 0.15) | 0.004 |
| Age group |  |  |
| 70 – 74 | REF | - |
| 75 – 79 | 0.37 (-0.15 – 0.89) | 0.17 |
| 80 – 84 | 0.28 (-0.38 – 0.94) | 0.40 |
| ≥ 85 | -0.27 (-1.29 – 0.76) | 0.61 |
| Female | 3.13 (2.67 – 3.60) | <0.001 |
| Income quintile |  |  |
| 1 – lowest | REF | - |
| 2 | -0.03 (-0.76 – 0.70) | 0.93 |
| 3 | -0.12 (-0.86 – 0.62) | 0.75 |
| 4 | -0.44 (-1.18 – 0.30) | 0.24 |
| 5 – highest | -0.60 (-1.34 – 0.13) | 0.11 |
| Rural residence | -1.57 (-2.30 – -0.84) | <0.001 |
| High comorbidity burden | 3.70 (3.23 – 4.17) | <0.001 |
| Frailty | 1.18 (0.24 – 2.12) | 0.01 |
| Cancer type |  |  |
| GI | -6.03 (-6.66 – -5.40) | <0.001 |
| GU | -5.25 (-6.17 – -4.32) | <0.001 |
| BP | Ref | - |
| Stage |  |  |
| 1 | Ref | - |
| 2 | 1.17 (0.33 – 2.00) | 0.006 |
| 3 | 1.68 (0.87 – 2.49) | <0.001 |
| 4 | 4.28 (3.32 – 5.23) | <0.001 |
| Missing | 3.92 (2.91 – 4.93) | <0.001 |
| Neo-adjuvant therapy | 0.88 (-0.15 – 1.92) | 0.09 |
| Change in one year | -0.20 (-0.27 – -0.12) | <0.001 |

p-value for differences in trajectories between patients with and without an unexpected ICU admission: p<0.001

**B: Sensitivity analysis estimating the impact of mechanical ventilation on the trajectory of overall symptom distress scores among patients with an unexpected ICU admission**

|  | Adjusted Beta coefficient (95% CI) | p-value |
| --- | --- | --- |
| Time (change per month) | -0.66 (-1.64 – 0.33) | 0.19 |
| Quadratic function of time (change per month^2^) | 0.05 (-0.05 – 0.16) | 0.33 |
| Receipt of mechanical ventilation | 0.56 (-2.11 – 3.23) | 0.68 |
| Interaction between time and receipt of mechanical ventilation | -0.38 (-1.58 – 0.82) | 0.53 |
| Interaction between quadratic function of time and receipt of mechanical ventilation | 0 (-0.13 – 0.13) | 0.95 |
| Age group |  |  |
| 70 – 74 | REF | - |
| 75 – 79 | 0.61 (-1.30 – 2.51) | 0.53 |
| 80 – 84 | 1.45 (-0.85 – 3.74) | 0.22 |
| ≥ 85 | -0.02 (-3.73 – 3.69) | 0.99 |
| Female | 2.66 (0.95 – 4.37) | 0.002 |
| Income quintile |  |  |
| 1 – lowest | REF | - |
| 2 | 1.89 (-0.75 – 4.54) | 0.16 |
| 3 | 1.59 (-1.08 – 4.26) | 0.24 |
| 4 | 0.63 (-2.03 – 3.29) | 0.64 |
| 5 – highest | 2.91 (0.31 – 5.50) | 0.03 |
| Rural residence | -2.46 (-5.10 – 0.18) | 0.07 |
| High comorbidity burden | 3.45 (1.76 – 5.14) | <0.001 |
| Frailty | 1.62 (-1.28 – 4.52) | 0.27 |
| Cancer type |  |  |
| GI | -5.21 (-7.42 – -3.01) | <0.001 |
| GU | -1.38 (-5.14 – 2.38) | 0.47 |
| BP | Ref | - |
| Stage |  |  |
| 1 | Ref | - |
| 2 | 0.55 (-2.77 – 3.87) | 0.75 |
| 3 | 0.83 (-2.47 – 4.14) | 0.62 |
| 4 | 1.59 (-2.12 – 5.30) | 0.40 |
| Missing | 2.29 (-1.42 – 6.00) | 0.23 |
| Neo-adjuvant therapy | -1.84 (-5.03 – 1.34) | 0.26 |
| Change in one year | -0.12 (-0.40 – 0.16) | 0.41 |

p-value for differences in trajectories between ICU patients with and without mechanical ventilation: p=0.99

**C: Sensitivity analysis estimating the trajectory of the change in overall symptom distress scores relative to pre-operative status**

|  | Adjusted Beta coefficient (95% CI) | p-value |
| --- | --- | --- |
| Time (change per month) | -0.89 (-1.21 – -0.57) | <0.001 |
| Quadratic function of time (change per month^2^) | 0.06 (0.02 – 0.09) | 0.002 |
| Unexpected ICU admission | 0 (-2.34 – 2.34) | 1.00 |
| Interaction between time and unexpected ICU admission | 0.02 (-1.01 – 1.05) | 0.96 |
| Interaction between quadratic function of time and unexpected ICU admission | -0.01 (-0.12 – 0.10) | 0.89 |
| Age group |  |  |
| 70 – 74 | REF | - |
| 75 – 79 | 0.77 (-0.31 – 1.85) | 0.16 |
| 80 – 84 | 0.34 (-1.10 – 1.77) | 0.64 |
| ≥ 85 | 0.22 (-2.22 – 2.66) | 0.86 |
| Female | -1.26 (-2.27– -0.25) | 0.01 |
| Income quintile |  |  |
| 1 – lowest | REF | - |
| 2 | 0.30 (-1.27 – 1.87) | 0.71 |
| 3 | 0.88 (-0.69 – 2.45) | 0.27 |
| 4 | 1.19 (-0.35 – 2.73) | 0.13 |
| 5 – highest | 1.21 (-0.34 – 2.75) | 0.13 |
| Rural residence | -0.09 (-1.77 – 1.59) | 0.92 |
| High comorbidity burden | 0.47 (-0.51 – 1.45) | 0.35 |
| Frailty | -3.26 (-5.21 – -1.30) | 0.001 |
| Cancer type |  |  |
| GI | -4.63 (-5.89 – -3.36) | <0.001 |
| GU | -3.36 (-5.14 – -1.57) | <0.001 |
| BP | Ref | - |
| Stage |  |  |
| 1 | Ref | - |
| 2 | -0.42 (-2.00 – 1.17) | 0.60 |
| 3 | 0.26 (-1.31 – 1.82) | 0.75 |
| 4 | 1.36 (-0.56 – 3.28) | 0.17 |
| Missing | -0.88 (-2.82 – 1.06) | 0.37 |
| Neo-adjuvant therapy | -0.27 (-1.57 – 1.04) | 0.69 |
| Change in one year | -0.11 (-0.29 – 0.07) | 0.23 |

p-value for differences in trajectories between patients with and without an unexpected ICU admission: p=0.26

**eTable 9. Pre-operative symptom burden**

|  | Unexpected ICU Admission | | Standardized difference |
| --- | --- | --- | --- |
|  | Yes  (n=375) | No  (n=3,345) |  |
| Pre-op ESAS score, median (IQR) |  |  |  |
| Pain | 0 (0-2) | 0 (0-2) | 0.01 |
| Tiredness | 3 (1-5) | 2 (0-5) | 0.21 |
| Drowsiness | 0 (0-3) | 0 (0-2) | 0.10 |
| Nausea | 0 (0-1) | 0 (0-0) | 0.23 |
| Lack of appetite | 1 (0-4) | 0 (0-3) | 0.29 |
| Shortness of breath | 0 (0-3) | 0 (0-2) | 0.19 |
| Depression | 0 (0-2) | 0 (0-2) | 0.14 |
| Anxiety | 2 (0-4) | 1 (0-4) | 0.07 |
| Poor wellbeing | 2 (1-5) | 2 (0-4) | 0.17 |
| Overall symptoms distress score | 14 (6-27) | 10 (4-22) | 0.24 |
| Moderate-to-severe pre-op symptoms, n (%) |  |  |  |
| Pain | 58 (15.5) | 470 (14.1) | 0.04 |
| Tiredness | 158 (42.1) | 1,114 (33.3) | 0.18 |
| Drowsiness | 87 (23.2) | 576 (17.2) | 0.15 |
| Nausea | 37 (9.9) | 169 (5.1) | 0.18 |
| Lack of appetite | 110 (29.3) | 704 (21.0) | 0.19 |
| Shortness of breath | 71 (18.9) | 467 (14.0) | 0.13 |
| Depression | 70 (18.7) | 450 (13.5) | 0.14 |
| Anxiety | 103 (27.5) | 847 (25.3) | 0.05 |
| Poor wellbeing | 125 (33.3) | 1,010 (30.2) | 0.07 |
| Overall symptoms distress score | 37 (9.9) | 241 (7.2) | 0.10 |

**eFigure 1. Cohort creation**


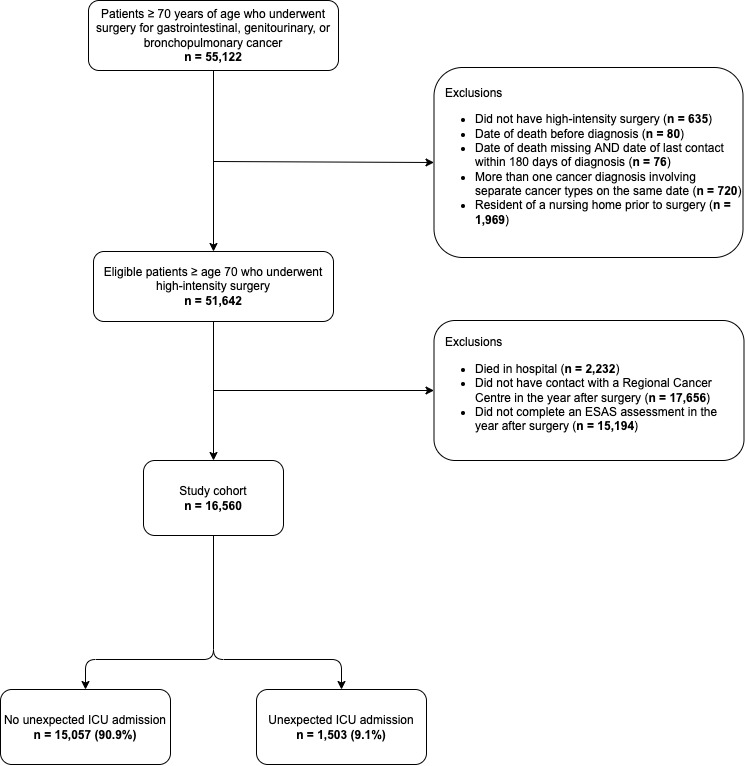


**eFigure 2. Unadjusted trends in the proportion of patients experiencing moderate-to-severe symptoms**

**a:** Total symptom score

**b:** Individual symptom scores

**i**: Pain **ii**: Tiredness

**iii**: Drowsiness **iv**: Nausea

**v:** Lack of appetite **vi:** Shortness of breath

**vii:** Depression **viii:** Anxiety

**ix:** Poor wellbeing

**eFigure 3. Trajectory of the probability of experiencing a moderate-to-severe burden of individual symptoms during the year after surgery, adjusted for baseline patient characteristics**

**a**: Pain **b**: Tiredness


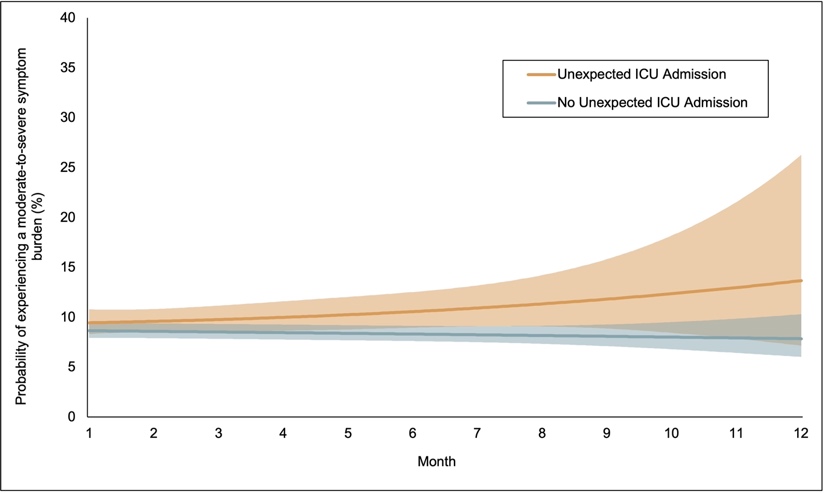

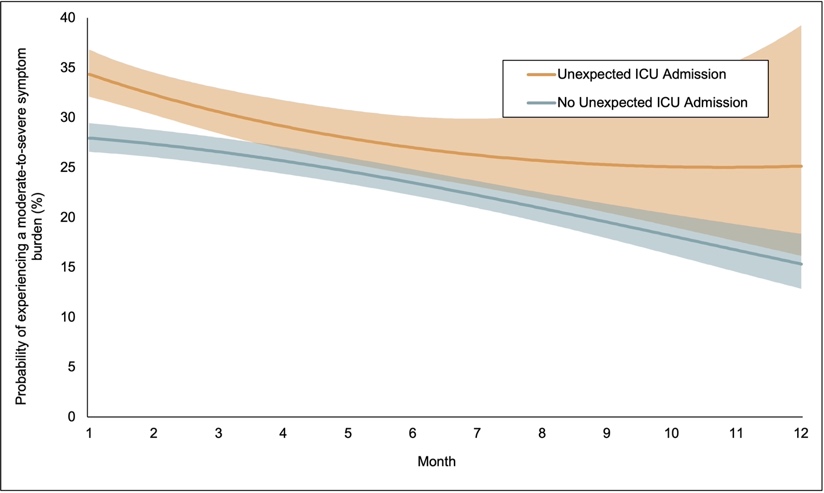


**c**: Drowsiness **d**: Nausea


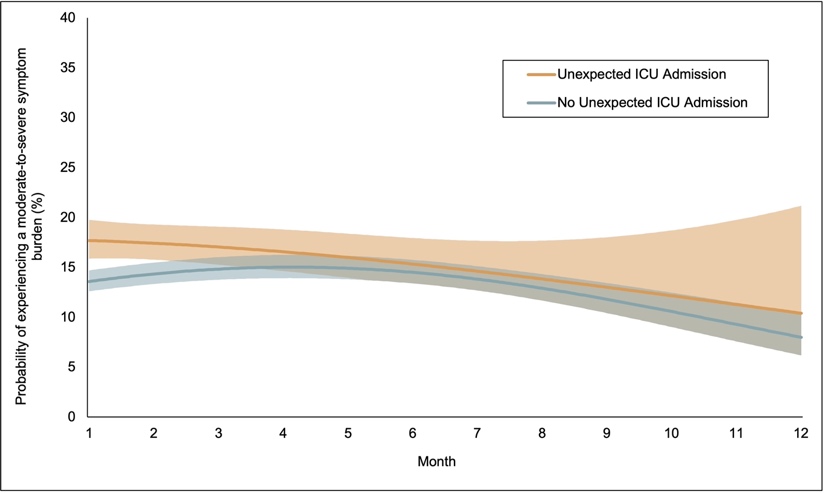

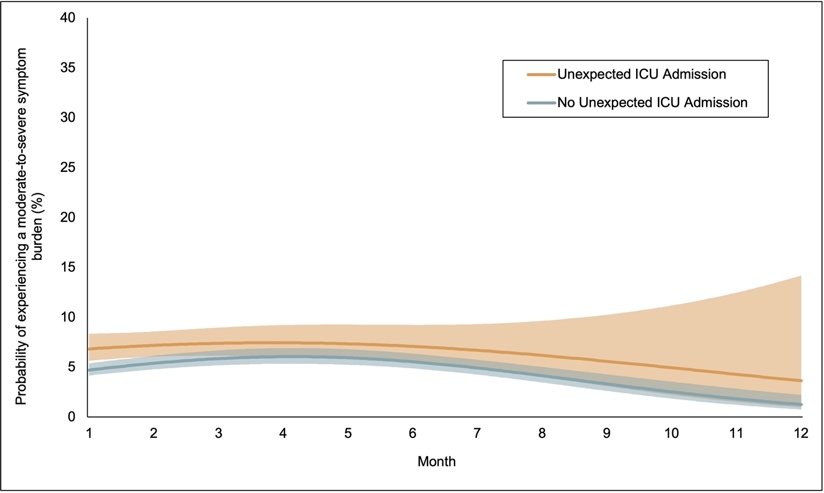


**e:** Lack of appetite **f:** Shortness of breath


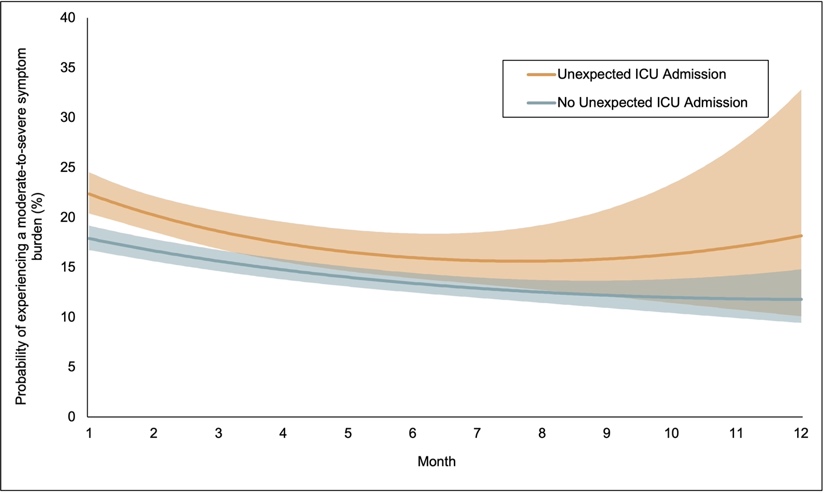

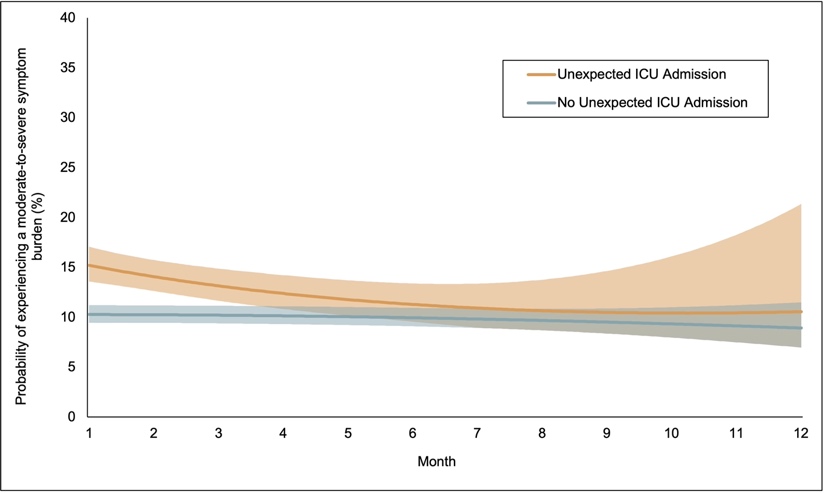


**g:** Depression **h:** Anxiety


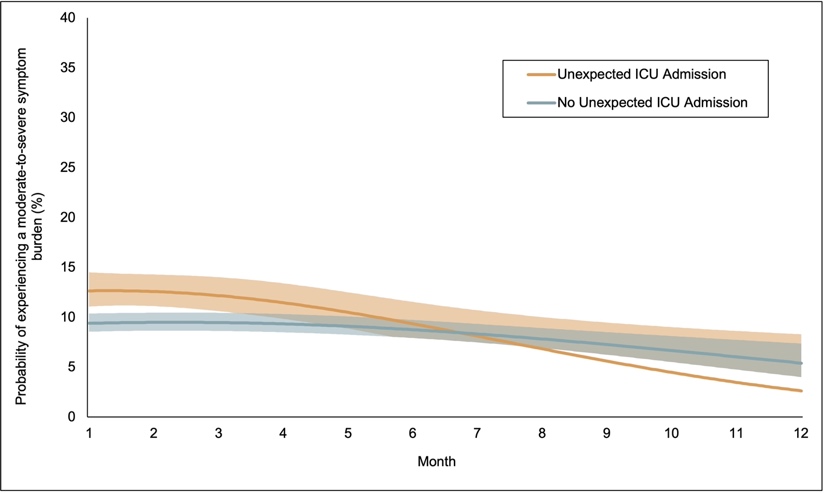

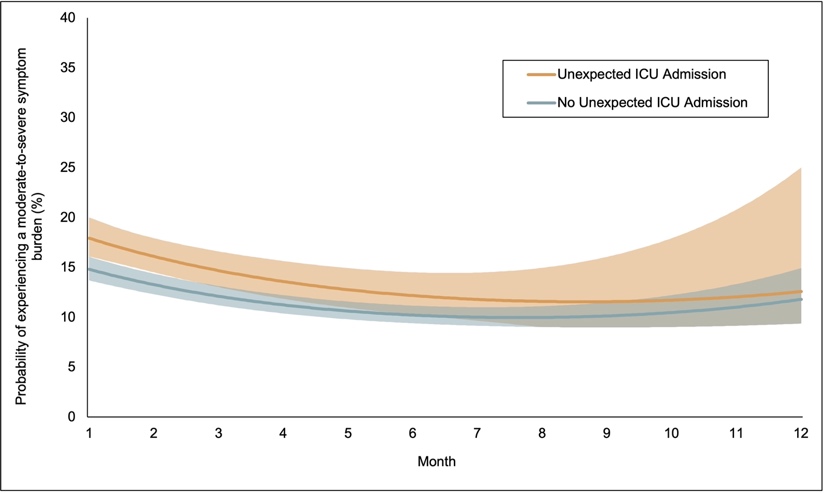


**i:** Poor wellbeing

**
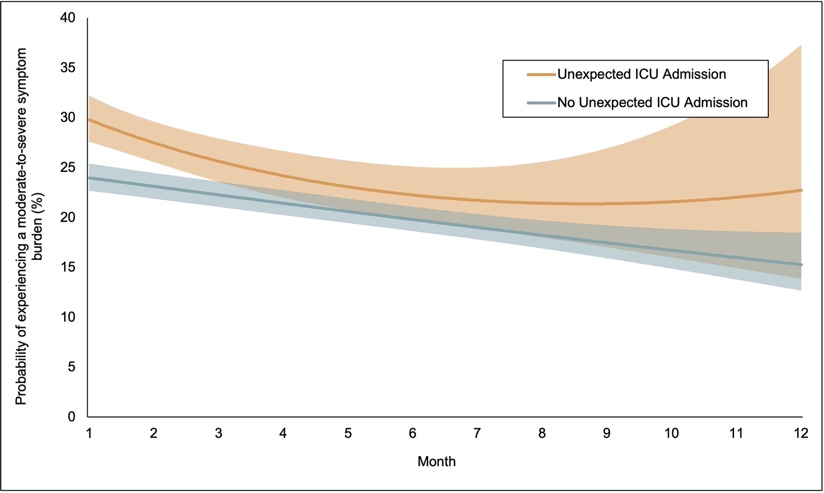
**

* The modified log-Poisson model used to create this figure was adjusted for age, sex, income quintile, geographic location, burden of comorbid illnesses, frailty, cancer site and stage, receipt of neo-adjuvant therapy, and year of diagnosis. The figures represent the average probability of developing a moderate-to-severe symptom burden for a male patient, age 70-74, with a low burden of comorbid illness, not identified as frail, diagnosed with stage one bronchopulmonary cancer in 2013, who did not receive neo-adjuvant treatment, and reside in an urban region in the lowest income quintile.

Note due to graphical limitations not all confidence intervals are able to be visually displayed

**eFigure 4. Trajectory of individual symptom burden during the year after surgery, adjusted for baseline patient characteristics**

**a**: Pain **b**: Tiredness

**c**: Drowsiness **d**: Nausea

**e:** Lack of appetite **f:** Shortness of breath

**g:** Depression **h:** Anxiety

**i:** Poor wellbeing

* The GLMM used to create this figure was adjusted for age, sex, income quintile, geographic location, burden of comorbid illnesses, frailty, cancer site and stage, receipt of neo-adjuvant therapy, and year of diagnosis. The displayed trajectories are for a male patient, age 70-74, with a low burden of comorbid illness, not identified as frail, diagnosed with stage one bronchopulmonary cancer in 2013, who did not receive neo-adjuvant treatment, and reside in an urban region in the lowest income quintile.

**eFigure 5. Sensitivity analysis examining the trajectory of the change in individual symptom burden during the year after surgery relative to pre-operative status**

**a**: Pain **b**: Tiredness

**c**: Drowsiness **d**: Nausea

**e:** Lack of appetite **f:** Shortness of breath

**g:** Depression **h:** Anxiety

**i:** Poor wellbeing

* The GLMM used to create this figure was adjusted for age, sex, income quintile, geographic location, burden of comorbid illnesses, frailty, cancer site and stage, receipt of neo-adjuvant therapy, and year of diagnosis. The displayed trajectories are for a male patient, age 70-74, with a low burden of comorbid illness, not identified as frail, diagnosed with stage one bronchopulmonary cancer in 2013, who did not receive neo-adjuvant treatment, and reside in an urban region in the lowest income quintile.
